# Leading the way together: a cluster randomised controlled trial of the 5R Shared Leadership Program in older adult walking groups

**DOI:** 10.1186/s12966-022-01297-x

**Published:** 2022-06-03

**Authors:** Katrien Fransen, Tegan Cruwys, Catherine Haslam, Peter Iserbyt, Jan Seghers, Julie Vanderlinden, Jannique van Uffelen, Elvire Verbaanderd, Filip Boen

**Affiliations:** 1 Department of Movement Sciences, KU Leuven, Tervuursevest 101, box 1500, 3001 Leuven, Belgium; 2 Research School of Psychology, The Australian National University, Canberra, Australia; 3 School of Psychology, The University of Queensland, St. Lucia, Australia

**Keywords:** 5R^S^, Group identification, Social identification, Peer leadership, Identity leadership, Elderly, Physical activity, Well-being, cohesion, Walking group

## Abstract

**Background:**

With a rapidly ageing society, healthy ageing has become a key challenge. Engagement in physical activity, and particularly walking, is a key strategy that contributes to healthy ageing amongst older adults. The purpose of the present study was to evaluate the efficacy of a group walking program for older adults that incorporates the 5R Shared Leadership Program (5R^S^). By implementing a structure of shared leadership and strengthening peer leaders’ identity leadership, 5R^S^ aims to cultivate a shared social identity amongst participants, which has in other contexts been associated with greater performance and well-being.

**Methods:**

A cluster randomised controlled trial was conducted to test the efficacy of the 5R^S^ group walking program on group identification, group cohesion, walking activity, and well-being, compared to a regular group walking program for older adults. Nineteen older adult walking groups (i.e., the clusters; *N* = 503; *M*_*age*_ = 69.23 years, *SD* = 6.68) all participated in a 12-week structured group walking program. Nine walking groups (*n* = 304) were randomly assigned to the intervention in which participants received the 5R^S^ program in addition to regular group walking.

**Results:**

5R^S^ was successful in strengthening the identity leadership qualities of the appointed peer leaders. Multilevel regressions showed that 5R^S^ succeeded in increasing group cohesion and walking activity to a greater extent than a regular group walking program, while participants’ group identification and well-being increased to a similar extent in both conditions. Furthermore, structural equation modelling revealed that group identification mediated the impact of peer leaders’ identity leadership on group cohesion and well-being (but not walking activity).

**Conclusion:**

By harnessing the capacity of the group and its peer leaders, the 5R^S^ program offers a promising intervention to engage older adults in physical activity.

**Trial registration:**

The study was retrospectively registered as clinical trial on 9 September 2021 (NCT05038423).

## Introduction

The world’s population is ageing. In their latest report, the United Nations [1] predicted that by 2050 the global population of adults aged over 65 years will almost double the one in 2019. It is therefore no surprise that ageing well and living in good health have become priorities for many nations [2]. In this regard, the World Health Organisation [3] listed physical activity as one of the key behaviours in promoting ‘healthy ageing’. Research shows that there are numerous benefits to older adults in engaging in physical activity; these range from physical health benefits (e.g., reduced risk of all-cause mortality) [4], to cognitive improvement [5], and enhanced mental well-being (e.g., reduced symptoms of anxiety and depression) [6]. However, despite these well-established benefits, a substantial proportion of older adults fail to meet the WHO recommendations for physical activity [3]. A key question, then, is how can we support and motivate older adults to increase and sustain their physical activity. 

When it comes to promoting physical activity among older people, walking has numerous advantages — it is the most natural form of physical activity that requires no special skills or equipment, it has a low risk of injury, and it is particularly suited for populations who are not sufficiently engaged in physical activity [7]. Indeed, a meta-analysis examining the effect of walking interventions among previously inactive adults found that increased walking led to enhanced fitness and a decrease in body weight, Body Mass Index, percentage body fat, and resting diastolic blood pressure [8].

### Walking Together: A Social Identity Lens

Walking in group, rather than individually, has become an increasingly popular strategy to promote physical activity. A systematic literature review found that interventions promoting group walking increased physical activity, and this effect was even stronger among those studies involving older adults [9]. Furthermore, group walking programs yield a wide-range of both physical and mental health benefits [10]. Interestingly, Kritz et al. [11] recently demonstrated that older adults walking with peers, compared to walking alone, experienced better health and motivational outcomes (e.g., improved physical activity, autonomous motivation, functional capacity, and reduced body fat). These results were confirmed in a 14-year longitudinal study, which found that group-based sport or exercise had greater benefits on long-term physical activity and life expectancy compared to engaging in sport or exercise alone [12]. Furthermore, older adults reported that exercise groups with age peers had the added advantage of reducing social isolation and loneliness [13]. As these data suggest, group-based exercise has the potential to deliver additional health and well-being benefits for older adults through their capacity of offering meaningful social group interaction.

A theoretical framework that can shed light on how social group-based activity promotes health is the social identity approach, which has recently been applied to health [14–16]. The *social identity approach to health* recognises the important role that people’s social group memberships — such as a walking group — play in supporting health behaviour and outcomes. This occurs through the capacity for group belonging to inform a person’s sense of self or identity (i.e., as ‘us’ members of the walking group). When being integrated into one’s social identity, groups gain the power to influence people’s thoughts, feelings, and behaviour (e.g., to engage in, and sustain regular and frequent walking), thereby also increasing access to a range of health-enhancing psychological resources (e.g., social support, increased perceived control, self-esteem).

There is now an established body of research showing that when these social identities, derived from group membership, are a positive source of influence, they reduce social isolation, increase engagement in healthy behaviour, and improve overall health and well-being [17, 18]. Speaking specifically to intervention, a recent meta-analysis by Steffens et al. [19] found that group-based interventions with the capacity to build social identity (e.g., as part of a treatment group or a physical activity group) had a moderate-to-strong positive effect on a range of health outcomes — such as well-being, depression, stress, and physical health. As this suggests, belonging to a physical activity group, and more importantly, *identifying* strongly as a member of that group, increases members’ participation and their physical activity levels, which then yields more general health benefits [20, 21]. It is noteworthy too, that some of these benefits arising from group belonging have been found to be more pronounced in older adults [22]. Indeed, given that older adults are more likely to live alone, lose family or friends, have chronic illnesses, or suffer from hearing loss, they are at increased risk for social isolation and loneliness [23]. Accordingly, there is potential for older adults to benefit even more than their younger counterparts from meaningful group-based intervention. This speaks to the primary aim of the current study; to determine whether the efficacy of walking group interventions for older people can be further enhanced by specifically targeting and building members’ strength of identification with others in their group. And, key to providing this test, is targeting an important factor known to underlie the development of such identification in interventions — group leadership.

### The Role of Peer Leaders in Cultivating a Shared Social Identity

Alongside research investigating the application of the social identity approach to the domain of health, is its application to understanding effective leadership [24]. A fundamental finding from the latter research is the central role that group leaders play in cultivating and strengthening a shared social identity within a group. While this line of research originated in the organisational context, in the last decade, an increasing number of studies have demonstrated the same effects of group leadership in the domain of sport and exercise. In sport teams, leaders’ capability to strengthen athletes’ identification with their sporting group has been associated with greater team effectiveness and enhanced athlete well-being [25–28]. Recent work in exercise settings, on the other hand, demonstrated that formal leaders were able to enhance members’ identification with their exercise group, and by doing so impacted their attendance rates, effort, and performance [29, 30].

While these are promising results, three important gaps in the literature are evident. First, the existing studies focus largely on younger or middle-aged adults who are physically active (i.e., sport team members or regular exercisers) [29–32]. Second, these studies have tended to target vigorous-intensity activity, such as a 5 km time trial on a cycling ergometer [33] or intensive group exercise classes [29]. Therefore, it is unknown whether these findings transfer to the engagement and maintenance of moderate-intensity physical activity (e.g., walking) among older adults. Third, these studies have only focused on formal group leaders in their examination of identity leadership. Yet, in sport contexts, it has been found that the coach is not the only driver of team identification [34]. Here, also peer leaders (i.e., athletes occupying a leadership role) have a critical role in cultivating a shared team identity, and by doing so impact the team’s confidence, its cohesion and effectiveness, as well as athlete well-being [27, 35–38]. Recent work suggests that the influence of peer leaders on team identification might even be greater than that of the coach [28]. While the promising impact of peer leaders on participants’ group identification has largely been ignored in exercise contexts, several studies illustrate the important role that peer leaders can occupy in exercise groups [39, 40]. Peer-delivered interventions were even found to be as effective as professionally-led interventions in increasing physical activity levels [41, 42].

Drawing on this previous literature, we argue that building a shared identity might be the key to increasing older adults’ engagement in physical activity, alongside health and well-being benefits. Furthermore, similar to previous findings in the sporting context, peer leaders might be promising drivers in cultivating the shared sense of ‘we’ and ‘us’ reflected in group identification. Therefore, in the present study, we aim to test the efficacy of an intervention in which we harness the capacity of peer leaders to cultivate a shared social identity in older adult walking groups, and do so using the 5R Shared Leadership Program – in short 5R^S^ [43].

### The 5R Shared Leadership Program (5R^S^)

The manualised 5R^S^ program, originally developed for organisations and sport teams, aims to enhance the leadership qualities of peer leaders by teaching them how to create, embody, advance, and embed a collective sense of ‘us’ in the groups they lead. More specifically, 5R^S^ consists of two key components that involve (1) implementing a structure of shared leadership, and (2) strengthening the identity leadership skills of the appointed peer leaders [43]. Identifying the best leaders in the team is an important first step in the implementation of an effective shared leadership structure. As the best leaders are often not the ones that already occupy formal leadership positions [44], Shared Leadership Mapping is used to identify who is best placed to perform four key leadership roles. These roles comprise those of (1) *task leader*, who provides task guidance and strategical advice; (2) *motivational leader*, who motivates team members to perform at their best; (3) *social leader*, who cultivates a positive team atmosphere; and (4) *external leader*, who represents the team to external agencies and stakeholders [43, 44].

**Fig. 1:**
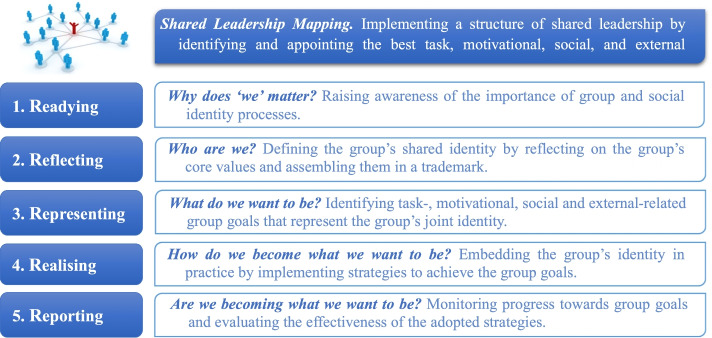
The 5R Shared Leadership Program, as developed by Fransen et al. [43]

After appointing these leaders, 5R^S^ aims to further develop the identity leadership skills of these peer leaders, by guiding them, along with their groups, through five program phases – known as the 5R’s, the content of which is visualised in Fig. 1 (for more details, see [43, 45]). In short, the groups are challenged to define their core values and uncover the shared identity that unites them. Next, they learn how to achieve their identity-related goals and aspirations by setting specific team goals. Thus, in addition to talking about ‘us’, the groups now also learn how to “walk the talk” and embed their identity in practice.

Previous studies in the sport context have found that this program strengthens peer leaders’ identity leadership and cultivates team identification [31, 32, 46]. Moreover, the 5R^S^ intervention yielded a range of other positive outcomes, including increases in athletes’ team confidence, access to social support from teammates, and well-being (i.e., improved health and reduced experience of burnout). However, as the program has never been evaluated outside of sporting and organisational contexts, its capacity to promote physical activity in the population of older adults is unknown.

### The present study

The primary objective of the present study was to implement an adapted 5R^S^ intervention in walking groups for older adults and evaluate its efficacy compared to a regular group walking program (i.e., comparison condition). Three associated aims were formulated.

Our first aim was to examine whether in this new context of older adults, 5R^S^ succeeded in strengthening the identity leadership skills of the appointed peer leaders. Here, we hypothesise that the perceived identity leadership quality of the peer leaders at the end of the 12-week program would be greater in the 5R^S^ condition compared to the comparison condition (H1).

Our second aim was to ensure that, although 5R^S^ focused on strengthening the quality of peer leadership, this would not be at the expense of the leadership qualities of the formal leader. In this vein, earlier research in organisation and sport contexts [47, 48] demonstrated that the opposite is true — teams with higher-quality peer leaders perceived their managers and coaches to be better leaders. We thus expect that in this context too, the increase in identity leadership qualities of the formal leaders in the 5R^S^ condition would be greater than those of the formal leaders in the comparison condition (H2).

The third and main aim of this research was to test the efficacy of 5R^S^ on group identification, group cohesion, well-being, and walking activity. Given previous evidence of the benefits of implementing a shared leadership structure and of cultivating identity leadership in other contexts, we hypothesised that the 5R^S^ program could yield additional benefits over and above those of a regular group walking program. More specifically, we expected that participants’ identification with their walking group (H3a), the group’s cohesion (H3b), participants’ well-being (H3c), and their walking activity (H3d) would all increase to a larger extent in the 5R^S^ condition compared to the comparison condition. We also hypothesised that participants would evaluate the walking program more positively when they had received 5R^S^ (H3e).

Our final aim was to examine a hypothesised mechanism through which 5R^S^ would enhance these outcomes; notably through group identification, which is expected to mediate the relationship between peer leaders’ identity leadership quality and the outcomes of cohesion, well-being, and walking activity (H4).

## Methods

### Participants and Design

Participants were recruited through OKRA SPORT+, which is a multisport federation that offers a variety of sport-oriented activities for people aged 55 or over. These activities are organised in smaller community-based meeting points. One of these sport-oriented initiatives was a multicomponent lifestyle program, including an evidence-based 12-week walking program [49], complemented by dietary advice and a muscle strengthening component. In our study, we focused on the 12-week group walking program, the main component of this lifestyle program, which aimed to enhance participants’ physical activity and aerobic fitness levels based on personalised walking schedules and weekly group walks organised by the community-based meeting points. To effectively organise these weekly group walks, volunteers were appointed as formal leaders in each registered meeting point. These volunteers (in both the intervention and comparison condition) completed a 1-day training course organised by OKRA SPORT+, which included guidance on project administration and information on the program content. After this 1-day training course, these formal leaders were responsible for recruiting new participants and organising the weekly group walks.

We conducted a cluster randomised controlled trial, in which the walking groups represented the clusters. While this design did not allow us to randomly allocate participants to the different groups (as the recruitment was locally organised by the community-based meeting points), we did randomly allocate the different walking groups to the experimental conditions. More specifically, while all walking groups engaged in a 12-week structured walking program, only half of them received the 5R^S^ program. With respect to the estimated sample size, G*Power 3 [50] suggested that in order to detect an effect of ${\eta}_p^2$ = .01, we needed at least 167 participants in each intervention condition (*N* = 344 in total) to detect a significant (condition X time) interaction effect with a power of .96 and an alpha of .05. Considering an expected drop-out rate of 15% (based on previous research conducting similar walking interventions with older adults; e.g., [11, 51, 52]). and an estimated average number of 20 participants in each cluster, we aimed to collect data of 20 clusters. This study was approved by the ethical committee of the first author’s university (G- 2019 021530). Informed consent was obtained from all individual participants included in the study. Participation was voluntary, participants could withdraw their participation at any time, and full confidentiality was guaranteed.

OKRA SPORT+ contacted 28 meeting points, 19 of which agreed to participate in our study (i.e., response ratio of 68%), with walking groups ranging in size from 8 to 63 participants. This strategy enabled the recruitment of a total sample of 503 older adults with an average age of 69.23 years (*SD* = 6.68). The majority of participants were female (72%) and retired (97%). While most participants were already member of OKRA SPORT+ (and might have been familiar with each other from previous sport-related activities), 25% of the participants were new to the organisation.^1^ To avoid any location bias, random assignment of the walking groups to the experimental conditions was stratified on the basis of region. Within this stratification, random allocation resulted in nine walking groups being designated to the 5R^S^ intervention condition (*N* = 304; *M*_*age*_ = 69.80; 71% female) and 10 walking groups to the comparison condition (*N* = 199; *M*_*age*_ = 68.35; 74% female).

**Fig. 2:**
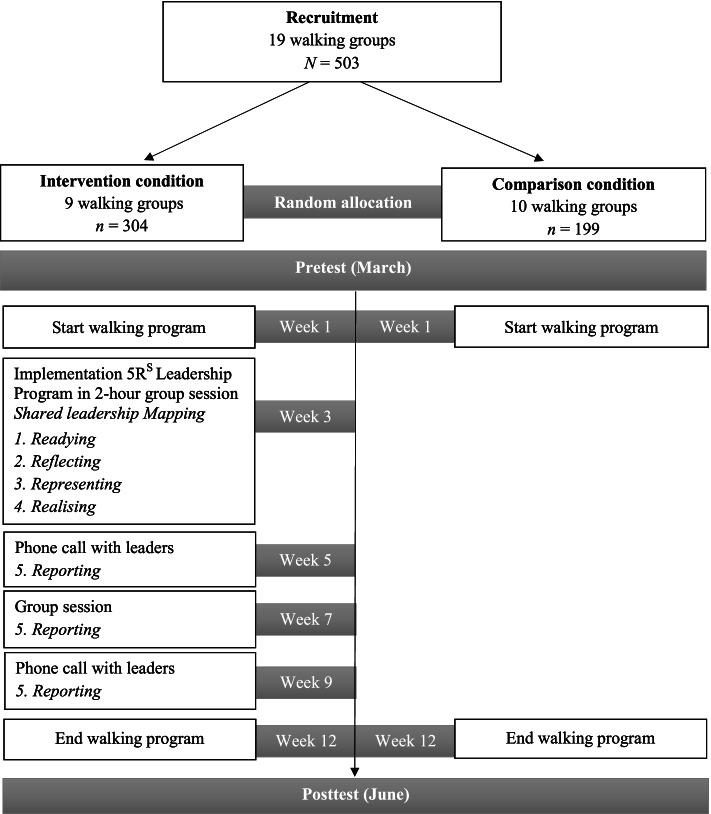
Study design and timeline

The timeline of the study is depicted in Fig. 2. The drop-out rate of the participants in the posttest (week 12) versus in the pretest (week 1) was 18.0% in the intervention condition and 20.1% in the comparison condition. These participants did not necessarily quit the walking program, but they did not attend the session in which the posttest was conducted.

#### The 5R Shared Leadership Program (5R^S^)

Participants in the intervention condition received the 5R^S^ program during the third week of the walking program. We opted for this delayed start of the intervention, as it was important for participants to get the chance to become acquainted with each other before engaging in the Shared Leadership Mapping exercise where they had to rate each other’s leadership quality. A researcher with knowledge of the program and its underlying theoretical assumptions guided the program by following a standardised script.

The content of the intervention resembled the original content of 5R^S^, as was described in the introduction [43]. However, in tailoring the 5R^S^ intervention to the specific setting context of walking groups for older adults, two important adaptations were made. First, given time restrictions, the core of the intervention (Readying, Reflecting, Representing, and Realising) was simplified in order to be delivered in a 2-hour group session. This entailed a reduction in the number of activities and the time spent on each activity. With respect to the Reporting phase, three different occasions were organised to get feedback of the participants and evaluate their progress towards the group goals. As visualised in Fig. 2, this was organised by two phone interviews with the leaders and one group session.

A second adaptation involved the use of a simplified version of the Shared Leadership Mapping. More specifically, only three leaders (task, motivational, and social leader) instead of four were appointed on their role. The reason was that in each walking group a volunteer was already appointed as formal leader whose role included the responsibilities of an external leader (i.e., handling the communication between the walking group and OKRA SPORT+). The role descriptions of the three leadership roles were tailored to the specific context, with (1) the *task leader* mapping the route of the weekly group walk together with the formal leader, and giving advice to the group members about the walking itself and about the developed task goals; (2) the *motivational leader* motivating the group members and encouraging them to motivate each other; and (3) the *social leader* creating a positive atmosphere in the walking group, welcoming new group members, and organising social events before or after the group walking session. Instead of rating all their team members’ leadership quality on these roles (which would take too much time in large walking groups), participants were asked to list their top 3 choices for the most suitable leader for each role. Based on this information, together with participants’ motivation to take up a certain leadership role, the researchers then identified and appointed a task, a motivational, and a social peer leader in every walking group. To reinforce their leadership role, each of these leaders received a captain’s arm band, which they wore during the walking sessions.

### Measures

All participants filled out a questionnaire at the start (pretest; week 1) and at the end of the walking program (posttest; week 12) including the questionnaires outlined below. Cronbach’s alphas, presented on the diagonal of Table 1, indicated good internal consistencies for all scales.

**Table 1:** Means, standard deviations, the number of identified extreme outliers that were removed, bivariate correlations, and Cronbach’s alphas for all the study variables

		*M*	*SD*	Extreme outliers	1	2	3	4	5	6	7	8	9	10	11	12	13	14
1	T1 Identity leadership formal leader	5.99	.86	3	(.94)													
2	T2 Identity leadership formal leader	6.22	.86	0	.39^***^	(.96)												
3	T1 Identity leadership extra peer leaders	5.37	1.19	0	.40^***^	.33^***^	(.96)											
4	T2 Identity leadership task leader	6.17	.77	1	.30^***^	.65^***^	.38^***^	(.97)										
5	T2 Identity leadership motivational leader	6.11	.85	0	.23^***^	.53^***^	.30^**^	.80^***^	(.98)									
6	T2 Identity leadership social leader	6.07	.89	0	.33^**^	.54^***^	.32^***^	.76^***^	.79^***^	(.98)								
7	T1 Group identification	5.51	1.05	2	.49^***^	.39^***^	.42^***^	.37^***^	.35^***^	.32^***^	(.90)							
8	T2 Group identification	5.81	.95	1	.25^***^	.50^***^	.35^***^	.48^***^	.53^***^	.58^***^	.45^***^	(.91)						
9	T1 Group cohesion	4.87	1.19	0	.38^***^	.29^***^	.50^***^	.34^***^	.37^***^	.38^***^	.63^***^	.36^***^	(.91)					
10	T2 Group cohesion	4.97	1.12	0	.15^*^	.30^***^	.41^***^	.46^***^	.43^***^	.50^***^	.39^***^	.56^***^	.58^***^	(.88)				
11	T1 Well-being	3.86	.54	1	.23^***^	.15^**^	.17^**^	.22^**^	.10	.11	.28^***^	.23^***^	.13^*^	.14^*^	(.93)			
12	T2 Well-being	3.93	.50	3	.23^***^	.23^***^	.23^***^	.22^**^	.17^*^	.16^*^	.32^***^	.32^***^	.26^***^	.19^***^	.70^***^	(.94)		
13	T1 Walking activity	864.98	856.70	14	−.07	.04	.02	.07	−.01	−.01	.08	.03	.03	.04	.12^*^	.10	a	
14	T2 Walking activity	1308.08	1016.25	12	.09	.07	.01	.01	.04	−.03	.14^*^	.13^*^	.10	.13^*^	.08	.15^*^	.19^**^	a

^*^p < .05; ^**^p < .01; ^***^p < .001. ^a^ As ‘Walking Activity’ was a single-item question, no Cronbach’s alpha could be calculated. Given that the data for walking activity were right-skewed, we also report the median scores, which were respectively 594 at T1 and 1188 at T2

#### Identity leadership

The 4-item Identity Leadership Inventory-Short Form [53] was used to evaluate the perceived identity leadership qualities of the different leaders. More specifically, the identity leadership of the formal leader was assessed in pre- and posttest of both conditions. Furthermore, given that no task, motivational, or social leaders were appointed at the time of the pretest, we asked participants in the pretest to rate the identity leadership of informal peer leaders in the team in general. At the posttest, we then asked participants in the intervention condition to rate the identity leadership of the appointed task, motivational, and social leader. All items were assessed on a 7-point Likert scale ranging from 1 (*strongly disagree*) to 7 (*strongly agree*).

#### Group identification

To assess participants’ identification with their walking group, we used the 4-item group identification measure (e.g., “I am glad to be a member of this walking group.”) [54]. The cognitive, evaluative, and affective aspects of identification were evaluated on a 7-point Likert scale ranging from 1 (*strongly disagree*) to 7 (*strongly agree*).

#### Group cohesion

Group cohesion was measured by the Group Environment Questionnaire [55]. For the purpose of this research, we focused on the two subscales assessing social cohesion, namely the individual attraction to the group (5 items, e.g. “Some of my best friends are in this walking group.”) and the group integration aspect (4 items; e.g., “Members of our walking group also engage in other activities together.”). The participants rated all items on a 7-point Likert scale ranging from 1 (*completely not agree*) to 7 (*completely agree*).

#### Well-Being

The Warwick-Edinburgh Mental Well-Being Scale was used to assess participants’ well-being [56]. It comprises 14 positively phrased items (e.g., “I’ve been feeling good about myself”) rated on a 5-point Likert scale ranging from 1 (*none of the time*) to 5 (*all of the time*).

#### Walking activity

The walking activity of the participants was assessed by the International Physical Activity Questionnaire – Short Form [57]. Participants indicated how many minutes per day and days per week they engaged in walking activity in the last 7 days. Next, these responses are weighted to simulate the required energy such that the continuous scores are expressed in metabolic equivalent (MET), with one MET representing a person's energy expenditure at rest. More specifically, the walking MET-minutes/week were calculated as 3.3*walking minutes*walking days [58].

#### General evaluation of the walking program

We also evaluated participants’ general perceptions of the walking program (i.e., the 12-week walking program in general, not specifically the 5R^S^ workshop) by asking them whether (1) their expectations of the program were fulfilled; (2) they felt more physically fit after the program; (3) they felt like continuing their walks after the program; (4) they would recommend the program to their peers. Participants rated these questions on a 5-point scale, including 1 (*not at all*), 2 (*barely*), 3 (*to a reasonable degree*), 4 (*to a large degree*), and 5 (*to a very large degree*).

### Data Analysis

First, extreme outliers (i.e., values at least three interquartile ranges below the first quartile or above the third quartile) were removed. Table 1 indicates the number of extreme outliers for each variable that were removed.

Second, to analyse the development of the identity leadership of the appointed task, motivational, and social leaders, we used pre-post paired samples t-tests as no leaders were appointed in the comparison group. As the leaders were not yet appointed in the pretest, we used the identity leadership of the informal peer leaders as baseline measure. Cohen’s *d* was reported as effect size and was calculated using a pooled standard deviation of the difference.

Third, to test the impact of the 5R^S^ intervention on the identity leadership of the formal leader, group identification, group cohesion, well-being, and walking activity, we conducted multilevel regression modelling in R [59], thereby accounting for the clustered nature of our data (i.e., participants belonging to walking groups). More specifically, we conducted 2 (time) X 2 (condition) within-between analyses with time as a Level 1-predictor, condition as a Level 2-predictor, and a random intercept for walking group at Level 3 to control for variability between the walking groups due to nesting of the data.

Finally, to examine the mediating role of group identification, structural equation modelling (SEM) was performed in MPlus [60], using robust maximum likelihood estimation method. To control for the nested structure of our data (i.e., participants being nested within walking groups), the MPlus command (type = complex) was used. This procedure adjusts the standard errors to prevent them from being inflated due to clustering [60, 61]. To reflect the changes over time, we used difference scores (posttest minus pretest) for all variables in the model. For the identity leadership of peer leaders, the mean of the identity leadership of task, motivational, and social leader at T2 was used as posttest value and the perceived identity leadership of the extra informal leaders at T1 was used as pretest value (since no peer leaders were appointed at that time). Given that only in 5R^S^ peer leaders were appointed, this model included data from the intervention group only. The fit of the model was evaluated using the Chi-square statistic (*χ*^*2*^), degrees of freedom (*df*), Comparative Fit index (*CFI*), the Tucker-Lewis index (*TLI*), the Root Mean Square Error of Approximation (*RMSEA*), and the Standardised Root Mean Square Residual (*SRMR*) [62].

## Results

The means and standard deviations for each variable along with bivariate correlations are shown in Table 1. Interesting to note is that the identity leadership of the task, motivational, and social leaders is significantly correlated with group identification, group cohesion, and well-being, which are in turn positively correlated with walking activity.

### Aim 1: Manipulation Check

**Fig. 3:**
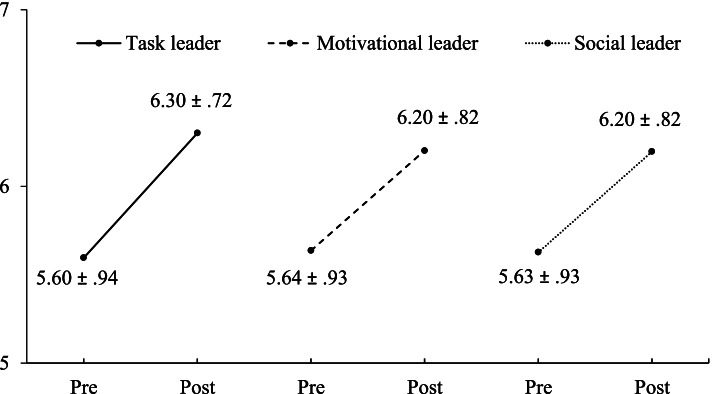
Results of the paired samples t-tests, comparing the identity leadership of the informal leaders at the pretest with the identity leadership of the task, motivational, and social leader at the posttest

The changes in identity leadership of the peer leaders in the intervention group over time are presented in Fig. 3. As the paired samples t-tests rely on the participants that completed both pretest and posttest measures, the baseline values vary slightly. In line with H1, a significant increase in identity leadership quality over time was observed for task leaders (*t* = 8.77, *p* < .001, *d* = .75), motivational leaders (*t* = 6.17, *p* < .001, *d* = .54), and social leaders (*t* = 6.40, *p* < .001, *d* = .56).

### Aim 2: Impact of 5R^S^ on the Leadership Quality of the Formal Leader

Our second aim was to examine whether empowering peer leaders in the 5R^S^ program did not come at the expense of the leadership quality of the formal leader. In line with H2, the opposite seemed to be true. More specifically, the multilevel regression (presented in Table 2) revealed a significant interaction, indicating that the identity leadership of the formal leader increased to a greater extent in the teams who received the 5R^S^ program, compared to those in the standard group walking program.

**Table 2:** Results of the multilevel regression modelling, including time as a level 1-predictor, condition as a level 2-predictor, and walking group as a level 3 random intercept. The table displays the means, standard deviations, time effects, and interaction effects (time x condition) for all outcome variables between intervention and comparison groups at T1 and T2

	*Intervention group*		*Comparison group*			
	*M (SD)* T1	*M (SD)* T2	*M (SD)* T1	*M (SD)* T2	*β*_*time*_ *(SE)*	*β*_*interaction*_ *(SE)*
Identity leadership of the formal leader	6.03 (.84)	6.39 (.78)	6.07 (.83)	6.15 (.81)	.23^***^ (.05)	.28^**^ (.11)
Group identification	5.69 (.89)	6.00 (.84)	5.50 (1.02)	5.66 (1.06)	.29^***^ (.06)	.15 (.12)
Group cohesion	5.07 (1.03)	5.35 (.90)	4.70 (1.22)	4.61 (1.12)	.11 (.06)	.47^***^ (.12)
Well-being	3.88 (.53)	3.97 (.49)	3.87 (.49)	3.93 (.45)	.08^***^ (.02)	.03 (.04)
Walking activity	774.50 (801.08)	1296.11 (1011.27)	1049.18 (975.44)	1252.70 (1071.84)	438.11^***^ (71.77)	296.36^*^ (143.64)

^*^p < .05; ^**^p < .01; ^***^p < .001

*Note.* The median scores of walking activity are 594 at T1 and 1188 at T2 in the intervention group; and 676.5 at T1 and 990 at T2 in the control group

### Aim 3: Impact of 5R^S^ on Identification, Cohesion, Well-being, and Walking Activity

**Fig. 4:**
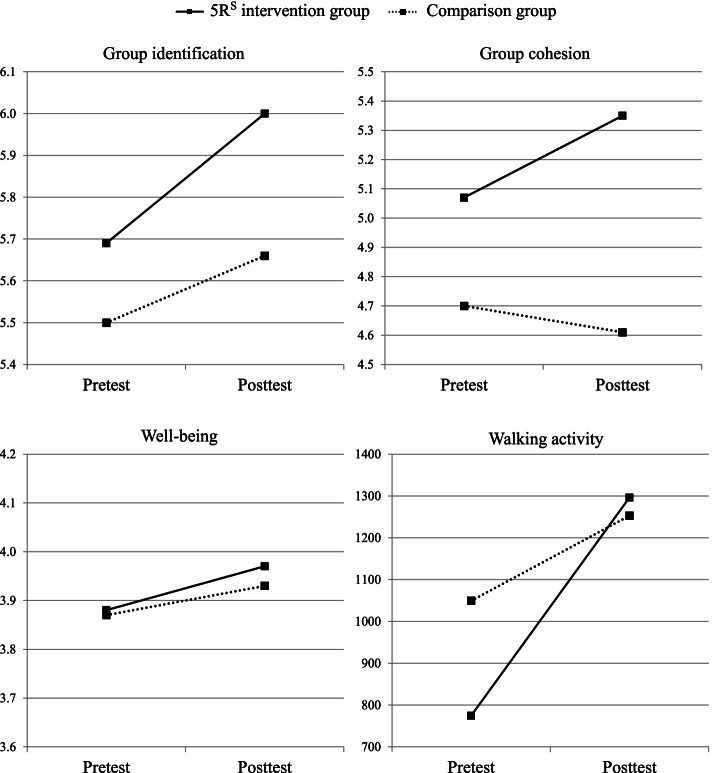
Pre- and postscores on the different outcomes for both the 5R^S^ intervention and comparison group

The results of the multilevel regressions can be found in Table 2 and are visually presented in Fig. 4. In line with H3b and H3d, we found significant interaction effects for group cohesion and walking activity. These interactions show that 5R^S^ produced additional improvements in group cohesion and walking activity, beyond those generated by a standard group walking program. However, in contrast with our hypotheses, no significant interaction effects were found for group identification and well-being. The significant time effects for these constructs indicate that the group walking program in itself significantly strengthened participants’ identification with their walking group and their well-being over time. It is nevertheless noteworthy that these increases in group identification and well-being were significant in the intervention group (*t* = 4.18, *p* < .001, *d* = .34 and *t* = 3.24, *p* = .001, *d* = .24), but not in the comparison group (*t* = 1.58, *p* = *ns*, *d* = .15 and *t* = 1.79, *p* = *ns*, *d* = .15).

Furthermore, the results in Table 3 demonstrate that participants who received 5R^S^ evaluated the walking program significantly more positively than those in the standard group walking program, which confirms H3e. More specifically, these participants felt that after the program their expectations were more fulfilled, they felt more physically fit, they were more eager to continue their walks, and were more likely to recommend the walking program to their peers, compared with those in the comparison condition.

**Table 3:** Results of the independent samples t-tests, comparing participants’ general evaluation of the walking program at T2 between participants in the intervention group and those in the comparison group

	Intervention group *M* (*SD*)	Comparison group *M* (*SD*)	*t*	*Cohen’s d*
My expectations of the program are fulfilled.	3.73 (.71)	3.54 (.73)	2.41^*^	.27
I feel fitter after the program.	3.47 (.76)	3.20 (.88)	2.98^**^	.33
I feel like continuing my walks after the program.	3.91 (.84)	3.72 (1.00)	1.97^*^	.21
I would recommend this program to my peers.	4.29 (.68)	4.13 (.79)	1.98^*^	.22

^*^p < .05; ^**^p < .01; ^***^p < .001

### Aim 4: Group Identification as a Mechanism of Change

**Fig. 5:**
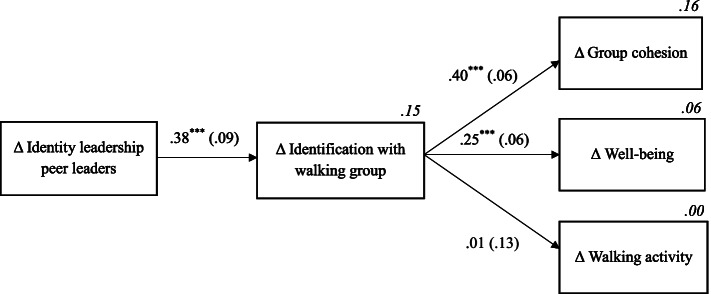
Structural equation model including the difference scores (Δ = posttest – pretest) of the identity leadership of the peer leaders, participants’ identification with their walking group, the group cohesion, participants’ well-being, and their walking activity. Standardised regression coefficients (with their standard errors) are depicted and the proportions of explained variance are presented in italics. ^***^p < .001

Our final aim was to test within the intervention group whether group identification mediated the relationship between changes in identity leadership of peer leaders over time and the associated changes in the different outcomes. The resulting model in Fig. 5 indicated an excellent fit to our data (*χ*^*2*^ = 5.55; *df* = 6; *p* = .48; *CFI* = 1.00; *TLI* = 1.02; *RMSEA* < .001; *SRMR* = .03). The results indicate that the increase in peer leaders’ identity leadership was positively related to an increase in participants’ identification with their walking group, which in turn predicted improvements in group cohesion and well-being. Based on the significant indirect effects (of increases in peer leaders’ identity leadership on increases in both group cohesion and well-being, as presented in Table 4) and the fact that the direct effects, when added to the model, were not significant, we can conclude that group identification fully mediated these relationships. However, contrary to our hypotheses, increases in group identification were unrelated to increases in walking activity.

**Table 4:** Standardised direct effects (DE) and indirect effects (IE) with according standard errors (SE) for the model represented in Fig. 3, as well as for the alternate models including different predictor variables. All the variables represent the difference scores (Δ = posttest minus pretest)

	*Δ Group identification*	*Δ Group cohesion*		*Δ Well-being*		*Δ Walking activity*	
	*DE (SE)*	*DE (SE)*	*IE (SE)*	*DE (SE)*	*IE (SE)*	*DE (SE)*	*IE (SE)*
Original model:							
*Δ* Identity leadership peer leaders	.38^***^ (.09)		.15^***^ (.05)		.10^***^ (.03)		.003 (.05)
*Δ* Group identification		.40^***^ (.06)		.25^***^ (.06)		.01 (.13)	
Model 2:							
*Δ* Identity leadership task leader	.32^**^ (.10)		.12^*^ (.05)		.08^**^ (.03)		.004 (.04)
*Δ* Group identification		.38^***^ (.06)		.24^***^ (.06)		.01 (.13)	
Model 3:							
*Δ* Identity leadership motivational leader	.33^**^ (.11)		.13^*^ (.06)		.08^*^ (.03)		.003 (.05)
*Δ* Group identification		.40^***^ (.06)		.23^**^ (.07)		.01 (.14)	
Model 4:							
*Δ* Identity leadership social leader	.42^***^ (.09)		.17^**^ (.05)		.10^***^ (.03)		.006 (.06)
*Δ* Group identification		.40^***^ (.07)		.25^***^ (.06)		.02 (.14)	

The fit values of Model 2 were: *χ*^*2*^ = 4.11; *df* = 6; *p* = .66; *CFI* = 1.00; *TLI* = 1.09; *RMSEA* < .001; *SRMR* = .03

The fit values of Model 3 were: *χ*^*2*^ = 6.75; *df* = 6; *p* = .47; *CFI* = .98; *TLI* = .97; *RMSEA* = .03; *SRMR* = .04

The fit values of Model 4 were: *χ*^*2*^ = 5.28; *df* = 6; *p* = .51; *CFI* = 1.00; *TLI* = 1.03; *RMSEA* < .001; *SRMR* = .03

^*^p < .05; ^**^p < .01; ^***^p < .001

We also tested this model separately for each of the three leadership roles (i.e., task, motivational, and social leader). An excellent fit to our data was found for all three models. The fit values, the direct, and the indirect effects can be found in Table 4. The main findings are thus robust for each of the different leadership roles.

### Robustness of the Results

We should note that, although groups were randomised to condition, an independent samples t-test at the group level revealed that, by chance, the average size of the walking groups was higher in the intervention condition (*M* = 33.78, *SD* = 18.03) than in the comparison condition (*M* = 19.90, *SD* = 10.57; *t* = 2.07, *p* = .05, *d* = .95). Therefore, analyses were repeated controlling for group size and revealed that the findings of both the multilevel regressions and the structural equation modelling were very robust when controlling for group size. Furthermore, while the above results are based on the dataset omitting the extreme outliers, posthoc tests confirmed that findings were robust when using the original dataset (including all outliers) as well as the dataset in which all outliers (i.e., values at least 1.5 interquartile ranges below the first quartile or above the third quartile) were removed. These findings attest to the robustness of our findings.

## Discussion

Walking groups are actively encouraged by health professionals and government bodies as a way to increase physical activity levels in an ageing population. The aim of the present study was to test whether the efficacy of these walking groups could be further improved by implementing a structure of shared leadership and cultivating a shared social identity through the 5R Shared Leadership Program (5R^S^), which was tailored to the context of older adult walking groups.

The study findings demonstrated that 5R^S^ was successful in strengthening peer leaders’ identity leadership skills or, in other words, their abilities to cultivate a shared sense of social identity. These findings corroborate earlier results in a sporting context, indicating that 5R^S^ indeed does what it promises, namely teaching peer leaders how to become better identity leaders [31, 32].

As formal leaders sometimes fear that empowering peer leaders within the group might be at the expense of their own leader status, our second hypothesis tested whether this fear was justified. The opposite seemed to be true, as the perceived identity leadership of the formal leader increased to a greater extent in the walking groups who received the 5R^S^ program, compared to those in the comparison condition. These findings are in line with earlier work in the organisational context [47] and in the sport context [48], showing that teams with higher, compared to lower, peer leadership quality perceived their formal leader to be a better leader. These results might constitute an important motivator to encourage formal leaders of walking groups to harness and empower the leadership talents within their group.

### The Efficacy of 5R^S^

Our third aim was to test whether the 5R^S^ program was able to yield additional benefits on group identification, group cohesion, well-being, and walking activity, beyond those found in the standard walking program. Here, our hypotheses were only partially fulfilled. In line with our expectations, 5R^S^ succeeded in increasing the group cohesion and participants’ walking activity to a greater extent than the standard walking program. The fact that group cohesion did not increase in the comparison condition over time indicates that organised group walks alone were not enough to strengthen the group’s cohesion. Rather, implementing a structure of shared leadership and enhancing peer leaders’ identity leadership qualities seems to be essential to develop a stronger sense of connectedness among members of the walking group. This finding is critical as previous research in walking groups identifies group cohesion as an important predictor of exercise adherence over a 12-month period [63], and that a main source of dissatisfaction with walking groups comes from not feeling integrated in the group [64]. Furthermore, by further enhancing participants’ walking activity, 5R^S^ can help older adults to meet basic guidelines for physical activity in older age [65].

Contrary to our expectations, no significant interaction effect was found for group identification and well-being. This finding seems to contrast with findings from earlier work in the sport context [31, 32]. It is worth noting, though, that the active comparison group constitutes a conservative test of the hypotheses because these participants also engaged in a 12-week walking program in the context of a social group. The significant time effects in the entire sample indicated that participants’ group identification and well-being increased throughout the program, although these increases were only significant in the intervention condition. Walking groups in general can thus be a positive vehicle to develop group identification and well-being. Another potential explanation for the non-significant interaction is that participants’ average identification with their walking group was already high, and significantly higher than the perceived group cohesion at baseline (*t* = 12.64, *p* < .001, *d* = .67), leaving limited room for differential improvement.

Furthermore, with respect to the general evaluation of the walking program, our findings showed that participants who received 5R^S^, compared to those in the standard group walking condition, felt that their expectations were more fulfilled, felt more physically fit after the program, were more eager to continue their walks after the program, and were more likely to recommend the walking program to their peers. These subjective evaluations indicated that the delivery of 5R^S^ on top of the group walking program boosted the satisfaction of participants. This is an important outcome as there is good evidence to suggest that when people’s needs and expectations regarding behaviour change are met, they are satisfied with that change, and are more inclined to maintain those changes [66].

### Group Identification as a Mechanism of Change

Given that the strength of our comparison group and potential ceiling effects might have overshadowed significant interaction effects, the structural equation model offers insight in whether the predicted theoretical principles can explain differences within the experimental group. Results of structural equation modelling, as presented in Fig. 5, indeed indicate that the expected theoretical assumptions were largely confirmed and group identification acted as a vehicle through which peer leadership lead to beneficial outcomes. More specifically, increases in the identity leadership quality of the peer leaders predicted increases in participants’ identification with their walking group, which in turn predicted improved well-being and a stronger cohesion in the walking group. These findings are in line with previous literature indicating the positive impact of peer leaders’ identity leadership on group identification [25]. Furthermore, the mediating role of group identification is in line with a large body of research, showing how social identities constitute a ‘social cure’, both capable of promoting individual well-being [17] and group cohesion [20]. Although the effects on these variables following 5R^S^ and standard walking were equivalent, the structural equation model did outline that intervention groups who experienced a growth in their peer leaders’ identity leadership skills also reported a stronger increase in participants’ group identification, which in turn led to a higher well-being and a stronger group cohesion.

Nevertheless, changes in group identification were not significantly related to increases in walking activity. Although Table 1 indicated significant (albeit small) correlations between the walking activity at T2 and group identification, group cohesion, and well-being at T2, these relationships did not hold when looking at the difference scores in the intervention condition. We can thus conclude that while 5R^S^ had an additional impact on participants’ walking activity compared to a standard walking program, this increase could not be directly attributed to the identity leadership qualities of the peer leaders. Rather, it was the implementation of a shared leadership structure in itself (with the appointment of a task, motivational, and social leader) that was likely the key to engage older adults to increase their walking activity.

### Strengths, Limitations, and Future Research

While most studies on the efficacy of walking programs rely on a control group in which no intervention takes place or in which participants follow an individual walking program, an important strength of the study was the use of an active comparison condition in which participants engaged in a 12-week group-based walking program. While group walking had the expected positive (albeit non-significant) effects on various outcomes, 5R^S^ produced additional significant benefits in group cohesion and walking activity over and above the standard group walking program.

A second strength of our study is its large sample size, despite the fact that our target population of older adults can often be difficult to reach. This is where collaboration with a large organisation supporting older adults was particularly helpful. Nevertheless, we must stress that the limitation of this approach is it may lead to a sample that is not representative of the older adult population more generally. Notably, through their association with OKRA we know that all participants engaged voluntarily and constituted a healthy, active, and engaged population (e.g., 72% of our participants indicated they engaged in other exercise activities outside the walking program^2^). Thus, the relatively high baseline in walking activity might have limited the amount of improvement that participants could experience. Moreover, given that most participants (75%) already belonged to a meeting point and knew each other in advance, it is possible that participants’ identification was already relatively high at the start, which left little room for improvement. To tease out these issues further research is needed to replicate the study, particularly in insufficiently active or socially isolated samples of older adults, who are not acquainted with each other in advance. We believe that if these persons can be supported and engaged to participate, the effects of 5R^S^ might be even more substantial than those observed in our sample.

Relevant to sample representativeness, we should also note that most of our participants were female (i.e., 72%). General population differences, with woman living longer than men [67], might have contributed in part to our greater success in recruiting women. Participants’ activity preference, with females being more interested in engaging in walking groups, might have further contributed to the gender imbalance in our sample. Indeed, a systematic review found that more women than men walked for leisure [68]. Yet, when looking at the gender breakdown in the oldest age groups, this effect was reversed and it was found that with increasing age more men than women walked for leisure. Preference for walking in general can thus not account for the observed gender imbalance in our sample. However, it seems that while women prefer the social elements of walking groups, this may not be the case for men, who rather prefer to walk alone [69]. To ensure that also male older adults can harness the benefits of group-based belonging, future research might therefore use physical activity interventions that are more aligned with men’s interests and values (e.g., group programs linked to a sport organisation or club [70, 71]).

The limited duration of our intervention is both a strength and limitation of the present study. On the one hand, we have shown that even a very short intervention, of which the core only takes 2 hrs, can have a meaningful impact on group cohesion and walking activity. On the other hand, however, a more elaborate intervention, with more time to unfold the 5R phases, with more follow-up sessions with the entire walking group, and in which we can support and guide the appointed leaders more intensively, could have a stronger impact. This may particularly be necessary in more difficult to reach or vulnerable populations who may require more time or support to work with 5R concepts. Furthermore, future studies with a long-term follow-up might be more suited to identify the benefits of 5R^S^ over regular group walking programs.

Another limitation is that we did not record whether all participants attended the 5R^S^ group session in which the core of the intervention was delivered. Not taking part in this workshop may have limited participants’ feelings of ownership of the goals that were set, thereby limiting the impact of 5R^S^. We did, however, measure participants’ attendance at walking group sessions in the intervention condition (i.e., the total number of times that they were absent), and this was found to be negatively related with participants’ perceptions of the identity leadership quality of the formal leader (*r* = −.18; *p* < .05), the motivational leader (*r* = −.21; *p* < .01) and the social leader (*r* = −.18; *p* < .05), but not the task leader. Less clear is the direction of this relationship, namely whether stronger identity leadership increased attendance or whether higher attendance contributed to participants having more chance to experience the provided identity leadership. Future research is needed to clarify the direction of this relationship.

Finally, the present study was conducted in walking groups that already had a formal leader. Given the observed impact of the appointed task, motivational, and social leaders in building the group’s cohesion and sustaining their peers’ motivation to engage in walking, future research should examine whether the impact of 5R^S^ might even be more visible in walking groups without a formal leader.

### Practical Implications

Although participation in walking groups yields numerous physical and mental benefits [10], older adults often encounter barriers to participation (e.g., motivational issues, low fitness, lack of company) [72] and have difficulty sustaining activity once commenced or doing so alone. Previous research already highlighted that peer leaders are beneficial, and as effective as professional leaders, in promoting and maintaining older adults’ adherence to exercise programs [42, 73, 74]. By harnessing the power of the group and implementing a structure of shared leadership, 5R^S^ indeed succeeded in increasing walking group cohesion to a larger extent than that observed in a regular walking group. As one of the major reasons for older adults to engage in walking groups is to increase social interaction and limit loneliness [75], 5R^S^ might help in motivating older adults to engage in walking groups and to sustain their walking behaviour on the long term.

Furthermore, our findings highlight the beneficial effects of empowering the leadership talent in the group by appointing task, motivational, and social leaders. Peer volunteers have been shown to be a cost-effective alternative compared with formal leaders to promote physical activity behaviour in older adults [76]. Rather than appointing volunteers, we selected the leaders who had a broad support base in their walking group. In this way, we ensure effective leadership, with leaders who are even more motivated to fulfil their role as they know that their peers accept and expect their leadership [43].

With regard to the quality of peer leadership, previous studies identified a wide variety of characteristics describing the ideal peer leader of a walking group of older adults, including being competent in making decisions, setting the pace, and initiating the walk, being a good motivator and encouraging peers, and being empathic and mastering social skills [40]. Given that it is unlikely to find such a variety in leader attributes in one peer leader, appointing different persons in the roles of task, motivational, and social leader will help in harnessing the leadership talents in the group.

## Conclusion

While groups are increasingly used to deliver behaviour change interventions, these interventions are only rarely based on theory and research on social group processes [77]. Drawing on the central tenets of shared leadership theorising and the social identity approach, the 5R^S^ program has been successful in supporting behaviour change (i.e., increasing walking activity) and strengthening group cohesion to a greater extent than a regular group walking program. While 5R^S^ did not instigate an additional impact on group identification and well-being beyond those initiated by the walking program, we did find support for our theorised mechanism of change. More specifically, participants’ identification with the group mediated the impact of peer leaders’ identity leadership on well-being and group cohesion. The 5R Shared Leadership program thus constitutes a promising intervention to engage older adults in group-based physical activity by harnessing the capacity of the group and its leaders.

## Data Availability

The dataset that was analysed in the current study is available on the Open Science Framework repository, with https://doi.org/10.17605/OSF.IO/V6C2M (https://osf.io/v6c2m/).

## Footnotes

1 Independent t-tests revealed that our findings were similar for both existing and new members, thereby testifying that participants’ previous membership was not a confounding factor.

2 Post-hoc analyses revealed that these participants were equally divided across both experimental conditions (i.e., 71.8% in the intervention group, and 71.2% in the comparison group; *p* = ns). A one-way ANOVA revealed also no significant difference in this ratio across the different meeting points.
